# Correction to “Metastatic Ewing Sarcoma, Patterns of Care and Outcomes of Patients in a Real‐Life National Setting Over a Decade”

**DOI:** 10.1002/cam4.72136

**Published:** 2026-08-03

C. Ducrot , D. Dinart , M. Bonneau , et al., “Metastatic Ewing Sarcoma, Patterns of Care and Outcomes of Patients in a Real‐Life National Setting Over a Decade,” Cancer Medicine 15, no. 6 (2026): e71909, 10.1002/cam4.71909.42237063 PMC13240556

The co‐author's name has been corrected from Natacha Entze Werle to Natacha Entz‐Werle.

This has been corrected in the published article.

We apologize for this error.

